# Modulation of Selectin-Mediated Adhesion of Flowing Lymphoma and Bone Marrow Cells by Immobilized SDF-1

**DOI:** 10.3390/ijms150915061

**Published:** 2014-08-27

**Authors:** Elizabeth A. Hedges, Andrew D. Hughes, Jane L. Liesveld, Michael R. King

**Affiliations:** 1 Department of Biomedical Engineering, Cornell University, 203 Weill Hall, Ithaca, NY 14853, USA; E-Mails: eah99@cornell.edu (E.A.H.); adh83@cornell.edu (A.D.H.); 2 Division of Hematology/Oncology, Department of Medicine, University of Rochester Medical Center, Rochester, NY 14642, USA; E-Mail: jane_liesveld@urmc.rochester.edu

**Keywords:** **:** stromal-derived factor-1 (SDF-1), lymphoma, metastasis, circulating tumor cell, P-selectin, cell adhesion

## Abstract

The α-chemokine, stromal-derived factor-1 (SDF-1), has been linked to the homing of circulating tumor cells to bone. SDF-1 is expressed by bone microvascular cells and osteoblasts and normally functions to attract blood-borne hematopoietic stem and progenitor cells to marrow. It has been shown that treatment of cancer cells with soluble SDF-1 results in a more aggressive phenotype; however, the relevance of the administration of the soluble protein is unclear. As such, a flow device was functionalized with P-selectin and SDF-1 to mimic the bone marrow microvasculature and the initial steps of cell adhesion. The introduction of SDF-1 onto the adhesive surface was found to significantly enhance the adhesion of lymphoma cells, as well as low-density bone marrow cells (LDBMC), both in terms of the number of adherent cells and the strength of cell adhesion. Thus, SDF-1 has a synergistic effect with P-selectin on cancer cell adhesion and may be sufficient to promote preferential metastasis to bone.

## 1. Introduction

It is well known that specific cancers metastasize preferentially to specific secondary tissues. For example, prostate cancers typically spread to the bone, while colorectal cancers spread to the liver and lungs. Previous work indicates that the expression of organ-specific ligands in the vasculature of specific tissues may be responsible for this behavior [1,2]. The α-chemokine, stromal-derived factor-1 (SDF-1), is expressed by bone marrow stromal cells and osteoblasts and is essential for the homing and retention of hematopoietic stem cells in the bone marrow [3,4]. SDF-1 has been linked to the “homing” of leukemic cells and circulating tumor cells (CTC) to bone, which leads to metastasis in the bone [5,6]. SDF-1 has also been found to enhance the survival and anti-apoptosis of hematopoietic progenitor cells, suggesting that SDF-1 and its receptor, CXCR4, have an important role in hematopoietic cell regulation [3].

Previous studies have shown that the treatment of cancer cells with soluble SDF-1 results in a more aggressive phenotype, both in terms of adhesion to endothelium and transmembrane migration [7]. Recent work by Zepeda-Moreno *et al.* has identified SDF-1 as an activator of leukemic cell adhesion to endothelial monolayers [8]. Additionally, treatment of cells with SDF-1 can result in the activation of the integrins lymphocyte function-associated antigen-1 (LFA-1) and very late antigen-4 (VLA-4), directional chemotaxis, vascular endothelial growth factor (VEGF) secretion and matrix metalloprotease secretion [9,10,11,12]. Many leukemic and lymphoma cells express CXCR4 on their surface [3,8], and CXCR4 expression levels have been proven to be a predictor of overall survival and relapse-free survival in patients with acute myeloid leukemia (AML) [13].

Many studies have investigated the effects of SDF-1 on AML cell lines and involve the incubation of cells with soluble SDF-1 for significant periods of time. However, in the body SDF-1 is expressed on the endothelium of bone marrow, and it is not clear if circulating cancer cells that interact with SDF-1 can respond to this signaling while still in the bone vasculature. Cellular interaction with the endothelium is characteristically rapid, facilitated by tethering and rolling due to binding to selectin molecules. The model for this is the adhesion of leukocytes to an inflamed endothelium, wherein fast moving leukocytes bind to P-selectin and roll slowly along the endothelium, become activated, firmly adhere to the vessel wall by integrin activity and migrate into the injured tissue [14,15,16]. Peled *et al.* in an important study found that immobilized SDF-1 on a substrate coated with P-selectin and ICAM-1 increased the ability of CD34+ cells and T-cells to form stable adhesions and resist detachment at high shear stresses [17]. The process of adhesion to endothelium is routine for healthy leukocytes and hematopoietic stem cells; however, studies suggest that circulating cancer cells are able to follow the same adhesion cascade to invade healthy tissues [18,19,20].

We have previously developed a microscale flow system that mimics the adhesion characteristics of the inflamed postcapillary venule, composed of selectin molecules and target cell-specific antibodies [21]. This has been applied to the investigation of leukocytes, hematopoietic stem cells and various cancer cells [22,23,24,25]. Here, we investigate the influence of SDF-1 on the adhesion characteristics of AML cells, as well as low-density bone marrow cells (LDBMC). Cell adhesion assays were performed on surfaces coated with P-selectin in addition to a range of concentrations of SDF-1 under flow in a microtube device. These assays were used to characterize the effects of SDF-1 on cell rolling and adhesion *in vitro*.

## 2. Results and Discussion

### 2.1. U937 and KG1a Express PSGL-1 on the Cell Surface

**Figure 1:**
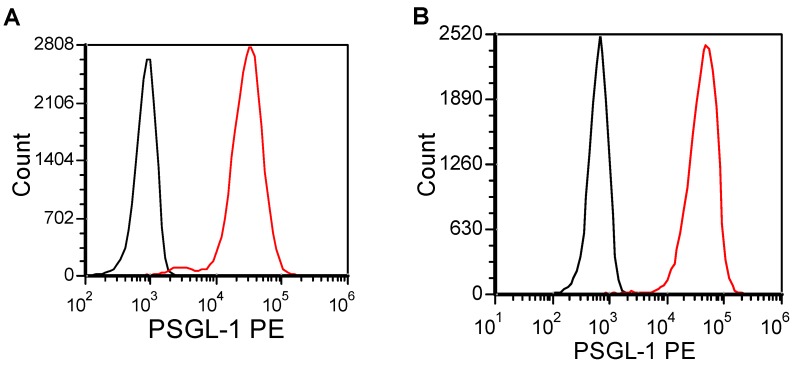
Flow cytometry was performed on lymphoma and leukemia cell lines to measure PSGL-1 expression. U937 (**A**) and KG1a (**B**) express high levels of PSGL-1. Red traces indicate PSGL-1 staining, while black traces indicate isotype control.

Previous studies have shown that AML cell lines express P-selectin glycoprotein ligand-1 (PSGL-1) on the cell surface [26,27]. The expression of the PSGL-1 on the surface of U937 and KG1a cells was confirmed using flow cytometry analysis (Figure 1). Both cell lines express significant quantities of PSGL-1 on their cell surface, suggesting the ability to bind to endothelial P-selectin under flow.

### 2.2. Differential Localization of CXCR4 Expression in U937 and KG1a

**Figure 2:**
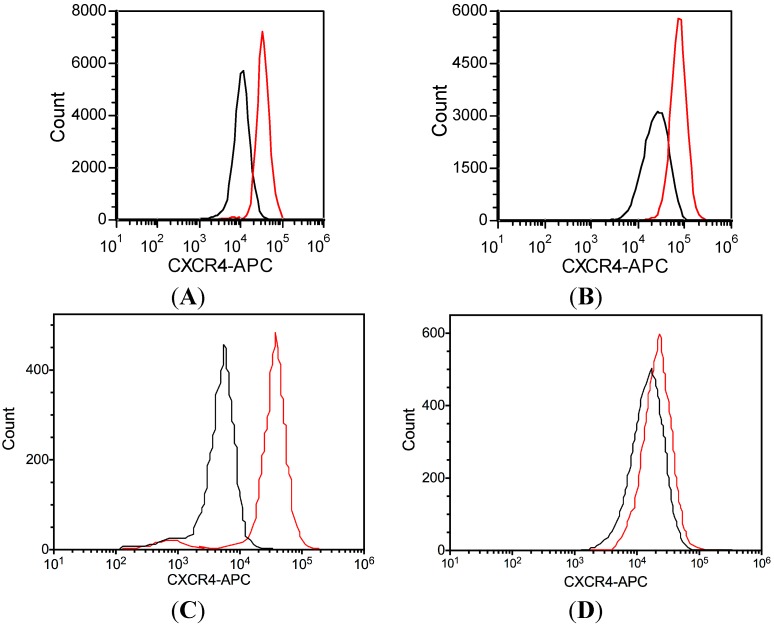
Cellular expression of CXCR4 was analyzed by flow cytometry. Two cell lines were investigated, and the location of receptor expression was determined as interior or exterior, following cellular permeabilization. Both U937 and KG1a cells were positive for CXCR4 expression when permeabilized (**A**,**C**, respectively); however, U937 cells were positive for CXCR4 expression when not permeabilized, while KG1a did not show external expression of CXCR4 (**B**,**D**, respectively). Red traces indicate CXCR4-1 staining, while black traces indicate isotype control.

Both U937 and KG1a cells are known to express CXCR4, the receptor for SDF-1. However, previous studies have not examined the localization of CXCR4 in the cell lines [7]. CXCR4 staining was compared between intact and permeabilized cells. Equivalent CXCR4 staining was observed on permeabilized and intact U937 cells, suggesting that CXCR4 is primarily expressed on the exterior of the cells (Figure 2A,B). KG1a cells were also positive for CXCR4 expression when permeabilized (Figure 2C). However, flow cytometry of intact KG1a was negative for CXCR4 expression, suggesting that this receptor is internalized in this cell type (Figure 2D).

### 2.3. P-Selectin and SDF-1 Have a Synergistic Effect on Rolling Velocity

**Figure 3:**
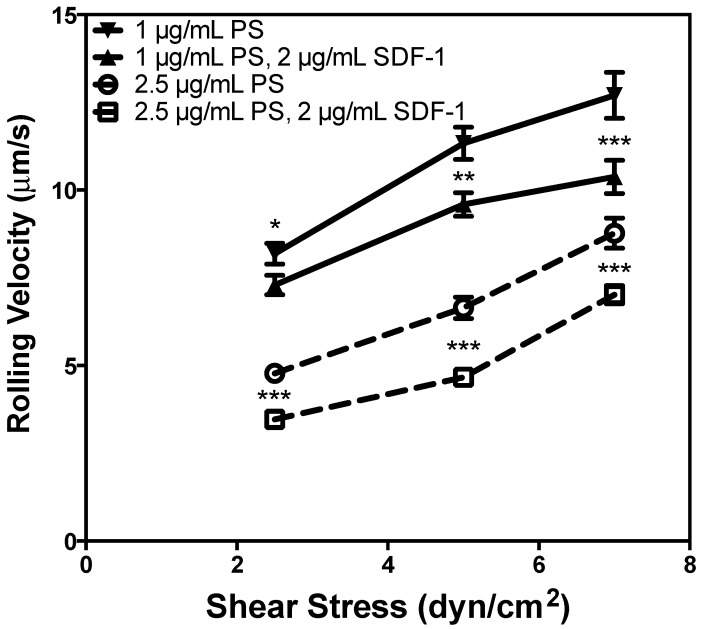
The rolling velocity of U937 cells was measured to determine the impact of immobilized stromal-derived factor-1 (SDF-1) on cell adhesion over a range of physiologically relevant shear stress. *N* = 3. * *p* < 0.05, ** *p* < 0.01, *** *p* < 0.001. Error bars represent standard error of the mean (SEM).

Many studies have examined CXCR4 expression in AML cells and the importance of CXCR4 in cell homing; however, to date, no study has shown the effect of immobilized SDF-1 on the adhesion of AML cells to selectins. The significance of SDF-1 on the modulation of the adhesion of hematopoietic cells has been established [17,28,29], suggesting a robust system. Thus, similar observations with cancer cells would lend import to such adhesion pathways in the disruption of metastasis. The average rolling velocity of U937 cells on surfaces coated with P-selectin alone was compared to the average rolling velocity of cells on surfaces coated with P-selectin and SDF-1 to determine the impact of SDF-1. It was determined that cell rolling velocity was significantly diminished on surfaces coated with P-selectin and SDF-1 compared to the same concentration of P-selectin alone (Figure 3). No cell rolling was seen on the tube coated with 2 μg/mL SDF-1 in the absence of P-selectin (data not shown). These results suggest that P-selectin is necessary for cells to roll on the microtube and that SDF-1 enhances the effect of P-selectin. A reduction in rolling velocity is an indication of the increased strength of cellular adhesion and would increase the chance of cells to firmly adhere to the vessel lumen, a necessary precursor to extravasation. It is noted that cells were not pretreated with soluble SDF-1; therefore, the effect of SDF-1 on cellular adhesion is not likely to be one of altered cellular phenotype, but rather a more immediate, superficial effect. As it is evident that increased adhesion strength would coincide with increased cellular recruitment to the endothelium following extravasation, we have identified a possible malignant role for SDF-1 expressed by bone microvasculature. Pre-incubation of CD34+ progenitor cells has been shown to have no effect on adhesion to P-selectin- and ICAM-1-coated surfaces, while inclusion of SDF-1 on the surface significantly increased the adhesion strength and the rate of firm adhesion [17,28]. It is important to note that, based on the enhanced adhesion of U937 cells to P-selectin in the presence of SDF-1, there is a possibility that the role of SDF-1 on selectin adhesion strength—in addition to integrin adhesion—is an important factor in progenitor cell arrest.

### 2.4. Higher Concentrations of SDF-1 Reduce the Rolling Velocity of U937 Cells

**Figure 4:**
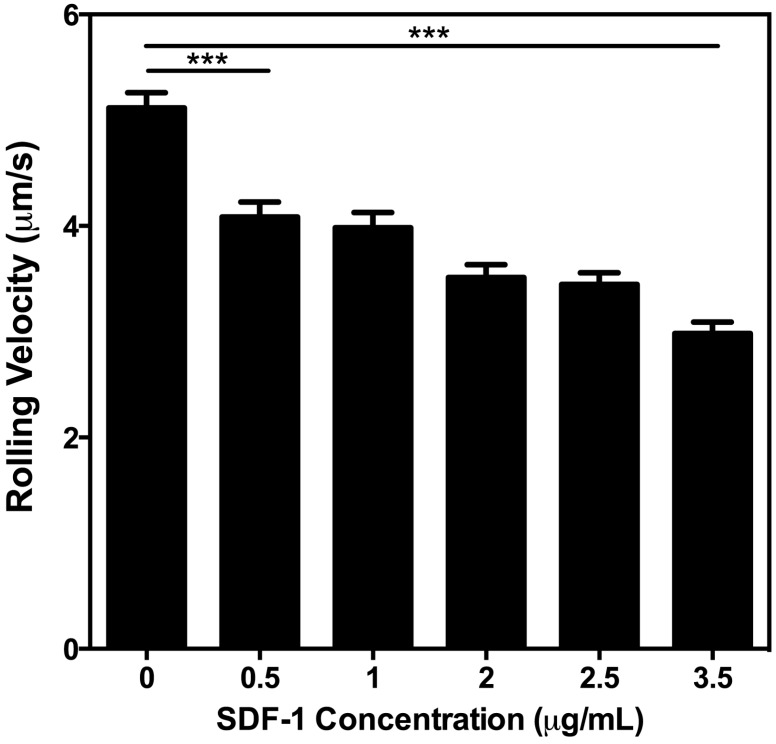
The rolling velocity of U937 cells was measured at a constant shear stress of 2 dyn/cm^2^. All surfaces were incubated with 2.5 μg/mL P-selectin while the concentration of SDF-1 was varied. *N* = 3. *** *p* < 0.001. Error bars represent SEM.

The effects of different SDF-1 concentrations on U937 cells were examined in a dose-dependent rolling assay. The rolling velocity of U937 cells was analyzed at a constant shear stress of 2 dyn/cm^2^ and a P-selectin concentration of 2.5 μg/mL, while the concentration of SDF-1 in each microtube was varied. Results demonstrate that higher concentrations of SDF-1 significantly reduce the rolling velocity of U937 cells (Figure 4). Rolling velocity significantly decreased with the addition of small amounts of SDF-1 and continued to decrease in a dose-dependent manner. P-selectin was required for cell rolling on the experimental device surface, while SDF-1 significantly modulated the strength of the adhesion of U937 cells, as indicated by the reduction in rolling velocity. This finding may be important in the context of cancer and metastasis, due to the fact that highly heterogeneous expression of surface proteins has been described [30], meaning that the adhesion process is a possible screening process for different subpopulations of circulating tumor cells. Previous studies into the adhesion of leukemic and other cancer cells to P- and L-selectin have shown similar behavior on E-selectin [31], which is expected based on the similarities in molecular structure. It is expected that the impact of SDF-1 on adhesion to these other selectins would have a qualitatively similar effect on adhesion strength, modulated to different levels of adhesion strength depending on the selectin; however, future work is necessary to confirm this.

### 2.5. Flux Increases with Increasing Concentrations of Immobilized SDF-1

**Figure 5:**
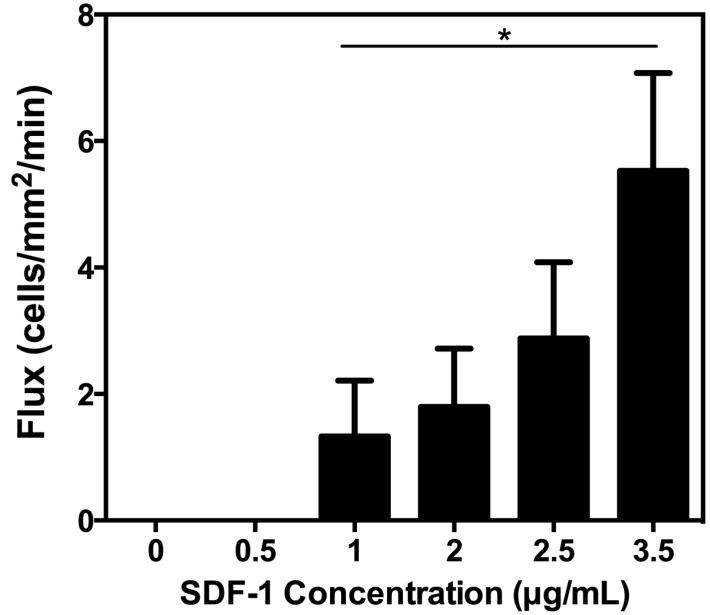
Cellular adhesion of U937 cells was measured in terms of flux at a constant shear stress of 2 dyn/cm^2^. Flux is defined here as the number of firmly adhered cells in a given area over time. Each surface was coated with 2.5 μg/mL P-selectin, while the concentration of SDF-1 was varied. *N* = 3. * *p* < 0.05. Error bars represent SEM.

An ancillary means of probing cellular adhesion is to measure the number of cells firmly adhered to an area of a surface for a given duration of time, which we have termed flux. This metric is a measure of the quantity of cells adhered to the surface regardless of rolling velocity. The number of U937 cells was analyzed at a constant shear stress of 2 dyn/cm^2^ and a P-selectin concentration of 2.5 μg/mL, while the concentration of SDF-1 in each microtube was varied. Rolling flux increased dramatically with increasing SDF-1 protein on the surface (Figure 5). No firm adhesion was seen in microtubes coated with 0.0 and 0.5 μg/mL, signifying that all adherent cells were exhibiting the weaker rolling adhesion behavior. Previously published findings have shown that normal progenitor cells exhibit stronger adhesion to surfaces on which they can engage selectin, as well as integrin ligands with the inclusion of SDF-1 [17]. Future investigations will determine the role of SDF-1 on cancer cell adhesion in the bone microvasculature, where there is the potential for SDF-1 to enhance adhesion to both selectin and integrin ligands, which would have important clinical implications.

### 2.6. SDF-1 Significantly Reduces the Rolling Velocity of LDBMC

**Figure 6:**
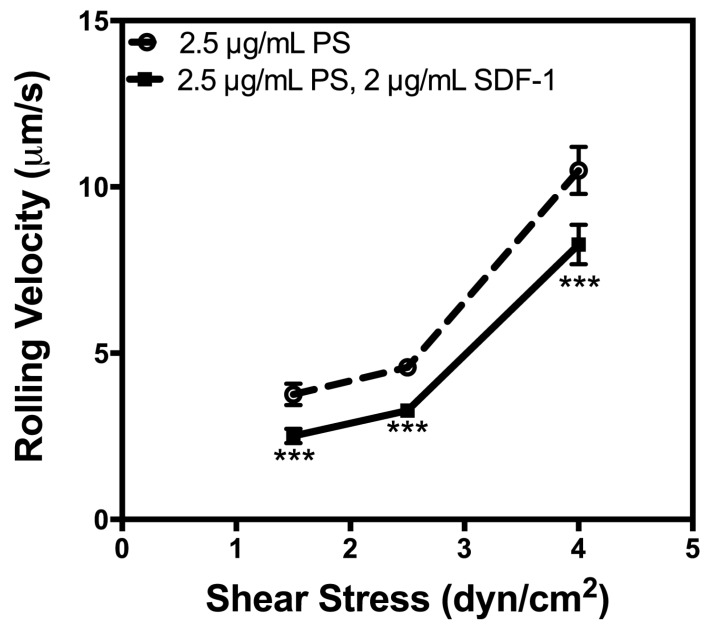
The impact of SDF-1 on the adhesion of low density bone marrow cells (LDBMC) from healthy donors was analyzed over a range of physiologically relevant shear stress. *N* = 3. *** *p* < 0.001. Error bars represent SEM. PS: P-selectin.

Many previous studies have demonstrated the role of SDF-1 in hematopoietic cell homing to bone marrow [3,4]. However, the effect of SDF-1 on adhesion to the endothelium by low density bone marrow cells (LDBMC) has not been previously examined. Given an equivalent surface concentration of P-selectin, SDF-1 significantly reduced the rolling velocity of primary LDBMC on the microtube surface compared to cells rolling on a surface coated with no SDF-1 (Figure 6). No cell adhesion was observed on the tube coated with 2.5 μg/mL SDF-1 alone. This result is quite similar to the synergistic effect observed with the U937 cell line (Figure 3). Thus, we have observed that there is both a healthy role for SDF-1 on the endothelial surface in recruiting LDBMC and a potential malignant role of SDF-1 in lymphoma by improving adhesion and therefore recruitment of lymphoma cells from blood flow, as well.

## 3. Experimental Section

### 3.1. Cell Culture

U937 and KG1a cells were purchased from ATCC (Manassas, VA, USA) and maintained in RPMI-1640 media (Gibco Laboratories, Grand Island, NY, USA) supplemented with 10% fetal bovine serum (FBS) (Atlanta Biologicals, Lawrenceville, GA, USA) and 1% penicillin-streptomycin (Sigma Chemical Co., St. Louis, MO, USA) in a humidified incubator at 37 °C with 5% CO_2_. Neither the RPMI-1640 media nor the FBS contained SDF-1.

### 3.2. Low Density Bone Marrow Cells

Normal low density bone marrow cells (LDBMC) were received from human donors after informed consent (University of Rochester Medical Center, Rochester, NY, USA).

### 3.3. CXCR4 and PSGL-1 Expression Studies

For the CXCR4 expression studies, U937 and KG1a cell lines were suspended at a density of 5 × 10^5^ cells/mL. To determine the external expression of CXCR4, cell lines were washed twice with Ca^2+^- and Mg^2+^-free Dulbecco’s Phosphate Buffered Saline (DPBS) (Gibco Laboratories, Grand Island, NY, USA). Cells were labeled with 20 μL anti-human CD184 conjugated to allophycocyanin (APC), clone 12G5, and a control sample was labeled with 20 μL of APC mouse IgG2a, k isotype, clone MOPC-173 (BioLegend, San Diego, CA, USA), and incubated on ice for 1 h. After incubation, cells were washed twice with DPBS; then, cells were analyzed using an Accuri C6 flow cytometer (Accuri Cytometers Inc., Ann Arbor, MI, USA), and plots were created using the FCS Express V3 (DeNovo Software, Los Angeles, CA, USA) software package. To determine the relative total expression of CXCR4, U937 and KG1a cells were suspended in 1.5 mL of methanol on ice for 20 min. The cells were then diluted with 8.5 mL DPBS. Cells were washed twice with DPBS containing 1% bovine serum albumin (BSA, Sigma Chemical Co., St. Louis, MO, USA). Cells were analyzed using flow cytometry as described above. External expression of PSGL-1 on the cell lines was conducted in the same manner as the external labeling of CXCR4 using PE mouse anti-human CD162 and PE mouse IgG1, k isotype control (BD Pharmingen, Franklin Lakes, NJ, USA).

### 3.4. Preparation of Microtubes

Micro-Renathane tubing (Braintree Scientific Inc., Braintree, MA, USA) with an inner diameter of 300 μm and an external diameter of 600 μm was cut to a length of 50 cm. The tubes were sterilized by drawing through 100 μL of 97% ethanol (VWR, West Chester, PA, USA) using an insulin syringe (Becton Dickinson, San Jose, CA, USA) followed by 100 μL of DPBS after 15 min. The tubes were prepared by drawing up 100 μL of 10 μg/mL Protein G in DPBS (Calbiochem, Philadelphia, PA, USA) and incubating for 1.5 h at room temperature (RT). One hundred microliters of P-selectin-IgG chimeric protein (R&D Systems Inc., Minneapolis, MN, USA) and anti-SDF-1 (Abcam, Cambridge, MA, USA) were drawn into the microtubes at varying concentrations and incubated at RT for 2 h. To immobilize SDF-1 on the microtube surface, tubes treated with anti-SDF-1 had 100 μL of soluble SDF-1 protein (Abcam, Cambridge, MA, USA) drawn through at double the concentration of the antibody, and tubes were incubated for 45 min at RT. Nonspecific cell adhesion was blocked by the incubation of 3% BSA in DPBS for 1 h. All tubes were washed with Ca^2+^-saturated DPBS.

The above process was repeated for the SDF-1 dose-response assay. The P-selectin IgG chimeric protein concentration was kept constant at 2.5 μg/mL. The anti-SDF-1 concentration was varied along with SDF-1 protein from 0 μg/mL SDF-1 to 3.5 μg/mL SDF-1. The SDF-1 concentration was double the concentration of anti-SDF-1 for all concentrations. All incubation times described above were maintained.

### 3.5. Microtube Flow Experiment

The coated microtubes were mounted on an inverted microscope, Olympus IX81 (Olympus America Inc., Melville, NY, USA), and connected to 5-mL syringes mounted on a multi-syringe pump (KDS 230, IITC Life Science, Woodland Hills, CA, USA). AML cell lines and LDBMCs were suspended at a concentration of 1 × 10^6^ cells/mL in calcium-saturated DPBS and Mg^2+^ Hank’s Balanced Salt Solution (HBSS) (Gibco Laboratories, Grand Island, NY, USA), respectively. Cells were perfused through the microtubes using the syringe pump at various physiological shear stresses: 1.5 to 7 dyn/cm^2^. Videos were recorded for 30 s at 3 random locations along the length of each microtube after cells were allowed to perfuse through for 5 min.

### 3.6. Data Acquisition

Videos of the rolling AML cells and bone marrow cells were recorded using a microscope-linked Hitachi CCD camera KP-M1AN (Hitachi, Japan) and a Sony DVD Recorder DVO-1000MD (Sony Electronics Inc., San Diego, CA, USA). DVD chapters were converted to 640 × 480 pixels at 29.97 frames per second using FFMPEGX (Fabrice Bellard, France). Rolling velocity, flux and cell adhesion were quantified using ImageJ (U.S. National Institutes of Health, Bethesda, MD, USA). Rolling cells were defined as any cell moving along the tube surface for greater than 2 s at a velocity less than 50% of the free stream velocity of a non-interacting cell near the wall of the tube. The number of firmly adhered cells was determined by counting the number of adhered cells in a measured area of the microtube in a 30-s period [32].

### 3.7. Statistical Analysis

Rolling velocity and number of adhered cells were plotted and analyzed using Prism 5.0b for Microsoft (GraphPad software, San Diego, CA, USA) as described previously [32]. ANOVA was used to compare differences in rolling velocity for each condition at each shear stress for the U937 cell rolling assay. A two-tailed *t*-test was used to determine significance for the U937 cell dose-response assay, U937 cell rolling flux and bone marrow rolling assay. *t*-tests were performed on the SDF-1 dose-response assay to determine the significance of rolling velocity between tubes treated with 0 μg/mL SDF-1 and 0.5 μg/mL SDF-1, as well as between 0 μg/mL SDF-1 and 3.5 μg/mL SDF-1. A two-tailed *t*-test was also performed to determine the significance of rolling flux between tubes treated with 1 μg/mL SDF-1 and 3.5 μg/mL SDF-1. A two-tailed *t-*test was performed on the bone marrow rolling assay to determine the significance of rolling velocity between the two conditions at each shear stress. Error bars represent the standard error of the mean (SEM). Three trials were performed for each experiment. The average rolling velocity or rolling flux was found for each trial, and the average velocities of the three separate experimental trials were averaged to produce the values for *N* = 3.

## 4. Conclusions

Differential localization of CXCR4 expression was observed on cell lines. This suggests that there will be dissimilar adhesive behaviors between cell lines and may predict differences in cancer aggressiveness. Cells with surface expression of CXCR4 are expected to adhere more strongly to the surfaces of bone marrow microvessels that co-express P-selectin and SDF-1. This is significant in that the ability of cancer cells to remain in the protective bone marrow niche correlates with increased cell survival and evasion of chemotherapeutics, leading to decreased patient survival. Immobilized SDF-1 present on the microtube surface significantly decreased the velocity of U937 cells rolling on selectin at a given P-selectin concentration, indicating stronger cellular adhesion, and SDF-1 was found to have a dose-dependent impact on adhesion strength. Immobilized SDF-1 on the microtube surface also significantly diminished the rolling velocity of LDBMC collected from healthy donors. Due to the fact that no cells were observed to be rolling or adhered on the surface coated with SDF-1 alone, the likely role of immobilized SDF-1 is to enhance a cell’s ability to adhere to a selectin-expressing surface. SDF-1 has an effect on adhesion strength, which may be sufficient to promote preferential migration to bone.
